# Rapid Simultaneous Determination of Three Synthetic Cannabinoids in Urine and Plasma of Rats Using Ultra-High Performance Liquid Chromatography Tandem Mass Spectrometry

**DOI:** 10.3390/toxics10100619

**Published:** 2022-10-18

**Authors:** Xing Ke, Yimei Tian, Dandan He, Pengqian Mu, Xuzhi Wan, Lange Zhang, Wei Jia, Qiao Wang, Yilei Fan, Yu Zhang

**Affiliations:** 1 Key Laboratory of Drug Prevention and Control Technology of Zhejiang Province, Department of Criminal Science and Technology, Zhejiang Police College, Hangzhou 310053, China; 2 National Engineering Laboratory of Intelligent Food Technology and Equipment, Zhejiang Key Laboratory for Agro-Food Processing, College of Biosystems Engineering and Food Science, Zhejiang University, Hangzhou 310058, China; 3 AB Sciex, Shanghai 200000, China; 4 School of Food Science and Engineering, Wuhan Polytechnic University, Wuhan 430023, China

**Keywords:** synthetic cannabinoids, 4-methylnaphthalen-1-yl-(1-pentylindol-3-yl) methanone, methyl (1-(5-fluoropentyl)-1H-indazole-3-carbonyl)-L-valinate, methyl 2-(1-(4-fluorobenzyl)-1H-indazole-3-carboxamido)-3-methylbutanoate, LC-MS/MS

## Abstract

Synthetic cannabinoids, a class of psychoactive compounds, are controlled as new psychoactive substances (NPSs) identified by the early warning system (EWS) of the European Monitoring Centre for Drugs and Drug Addiction (EMCDDA). At present, several new synthetic cannabinoids have appeared in the illegal drug market, including 4-methylnaphthalen-1-yl-(1-pentylindol-3-yl) methanone (JWH-122), methyl (1-(5-fluoropentyl)-1H-indazole-3-carbonyl)-L-valinate (5F-AMB), and methyl 2-(1-(4-fluorobenzyl)-1Hindazole-3-carboxamido)-3-methylbutanoate (AMB-FUBINACA). A convenient, rapid, and highly sensitive analytical method was developed to determine three synthetic cannabinoids in rat plasma and urine. The liquid chromatography tandem mass spectrometry (LC-MS/MS) method was optimized and validated to analyze the three synthetic cannabinoids in rat plasma and urine. The method identified intra-assay precision (1.3–9.0% and 2.8–6.7%), inter-assay precision (3.0–8.6% and 3.9–8.8%), limits of detection (0.003–0.004 ng/mL and 0.00125–0.002 ng/mL) and quantification (0.012–0.016 ng/mL and 0.003–0.005 ng/mL), recovery (95.4–106.8% and 92.0–106.8%) for rat plasma and urine, and the matrix effect (93.4–118.0%) for rat urine, and the correlation coefficients were above 0.99 in the linear range. The established LC-MS/MS method was successfully used to simultaneously detect the JWH-122 and 5F-AMB in rat plasma and JWH-122, 5F-AMB, and AMB-FUBINACA in rat urine. The present study provides methodological support for internal exposure assessment of three synthetic cannabinoids and promotes the quantitative analysis and technical supervision of synthetic cannabinoids.

## 1. Introduction

Synthetic cannabinoids as psychoactive substances can interact with endocannabinoid receptors (CB1 and CB2), which may induce anxiety, psychosis, hallucinations, tachycardia, and seizures [1,2]. Over the past decades, synthetic cannabinoids have been marketed via the internet as herbal incenses and room deodorizers [3]. In 1994, Huffman et al. synthesized the first synthetic cannabinoids, including naphthylmethylindoles, naphthoylpyrroles, naphthylmethylindenes, and phenylacetylindoles, structurally categorized into cyclohexylphenoles, phenylacetylindoles, benzoylindoles, and naphthoylindoles. Subsequently, naphthoyl indoles (JWH-018) increasingly arose, and so did indazoles (AKB-48) and indole carboxamides (MDMB-CHMINACA). During the past decade, synthetic cannabinoids, such as 4-methylnaphthalen-1-yl-(1-pentylindol-3-yl) methanone (JWH-122), have emerged rapidly in the illicit drug market as one of the main effective ingredients in spice drugs [4,5]. Methyl (1-(5-fluoropentyl)-1H-indazole-3-carbonyl)-L-valinate (5F-AMB), as the derivative of synthetic cannabinoids AB-PINACA and 5F-ABPINACA, was first reported as a constituent in herbal incenses on the Japanese drug market in 2014 [6,7]. Methyl 2-(1-(4-fluorobenzyl)-1H-indazole-3-carboxamido)-3-methylbutanoate (AMB-FUBINACA) is an indazole-based synthetic cannabinoid, which was firstly detected in America in 2014, and was included in the legal supervision list in China in 2018 [8,9]. Therefore, it is warranted to adjust analytical methods continuously to surveil the emerging synthetic cannabinoids.

The choice of biological matrices is critical for the analytical methods of various chemicals [10]. Plasma and urine samples are generally harnessed to analyze both synthetic cannabinoid parents and metabolites [11]. Synthetic cannabinoids can be identified via monitoring the parent of synthetic cannabinoids or their metabolites. Analysis of metabolites is difficult and costly, and thus it is appropriate to directly measure the parental synthetic cannabinoids [10]. Most synthetic cannabinoids are hard to detect due to the low concentrations in biological matrices. LC-MS/MS is used to identify and quantify parent synthetic cannabinoids owing to its high sensitivity and selectivity, but it is concurrently interfered by matrices [12]. Notably, the pretreatment methods, such as an appropriate extraction method of synthetic cannabinoids from plasma and urine matrices, can reduce ion suppression, attenuate matrix interference, concentrate the target synthetic cannabinoids, and increase sensitivity [13]. Protein precipitation has been subjected for extraction of synthetic cannabinoids in plasma owing to its high extraction efficiency and low levels of reagents [14]. Solid phase extraction is mainly harnessed for the separation, purification, and concentration of synthetic cannabinoids in blood and urine samples and can effectively remove matrix effects [15]. At present, the detection of synthetic cannabinoids mainly focuses on the main components in “Spices”, but scant research concerns the detection of synthetic cannabinoids in biological matrices [16]. Therefore, this study aimed to develop and validate a high-sensitivity method to detect synthetic cannabinoids including JWH-12, 5F-AMB, and AMB-FUBINAC in biological matrices, thus providing technical support for drug control.

## 2. Materials and Methods

### 2.1. Reagents, Supplies, and Specimens

JWH-122, 5F-AMB, and AMB-PUBINACA were purchased from Shanghai Research Institute of Criminal Science and Technology (Shanghai, China). Acetonitrile and methanol were obtained from Merck (Kenilworth, NJ, USA), and formic acid was purchased from ROE scientific INC (Kenilworth, NJ, USA). All solvents were HPLC grade. Water was purified with a Milli-Q system (Millipore, Bedford, MA, USA) and was used throughout the whole experiment, including sample treatment and instrument-based analysis. Blank rat plasma and urine samples were provided from Zhejiang Academy of Medical Science (Hangzhou, China). The solid phase extraction (SPE) column Oasis HLB (3cc/60 mg), Oasis WCX (3cc/60 mg), and Oasis MCX (3cc/60 mg) were purchased from Waters (Milford, MA, USA).

### 2.2. Animals and Treatment

The Sprague–Dawley male rats (n = 12, 6 weeks, weighing 190–210 g) were obtained from Zhejiang Academy of Medical Sciences (Hangzhou, Zhejiang, China) and acclimatized for 1 week before exposure in the experimental room (temperature 23 ± 2 ℃, humidity 55 ± 10%, 12 h light–dark cycle). After 1-week adaptation, rats were fasted for 16 h before oral administration. The rats were randomly allocated to 3 groups, the JWH-122, 5F-AMB, and AMB-PUBINACA group, while the doses were all 1 mg/kg bw/day by tail vein injection. The urine samples were collected at 2 h, 4 h, 8 h, and 24 h during the initial 24 h and every 24 h during the next 48 h. The blood samples were collected from the abdominal aorta. Finally, urine samples and blood samples were obtained from each rat and stored at −80 °C. All the above animal experimental procedures were approved by the Zhejiang Center of Laboratory Animals Welfare and Ethical Review Committee (Approval ID: ZJCLA-IACUC-20100005; Hangzhou, China).

### 2.3. Sample Preparation

In this experiment, extraction conditions of three synthetic cannabinoids in rat plasma and urine were optimized, and the detailed parameters are shown in Table 1. Briefly, 100 μL of plasma sample was transferred into a centrifuge tube with 100 μL of acetonitrile and 500 μL of methanol and then vortexed for 6 min. Finally, the supernatant was centrifuged at 12,000 rpm for 10 min then filtered through a 0.22 μm cellulose filter. Solid phase extraction (SPE) is a common pretreatment method for the synthesis of cannabinoids in urine [15]. The urine sample was extracted by SPE. The SPE cartridges were conditioned with 3 mL of methanol followed by 6 mL of water. Amounts of 1.6 mL of acetonitrile and water were added to 400 µL of urine, mixed uniform. After sample loading, the cartridges were respectively washed with 3 mL of 5% methanol, discard wash. The extract was eluted via 4 mL of methanol and evaporated to dryness under a stream of nitrogen at 40 ℃.

### 2.4. LC-MS/MS Analysis

The AB SCIEX ExionLCTM0AD XR high-performance liquid chromatograph system coupled with the SCIEX QTRAP 6500^+^ triple quadrupole/linear ion trap mass spectrometer system (AB SCIEX, Milford, MA, USA) was used for analysis. Chromatographic separation was carried out using a Waters UPLC HSS T3 (150 × 2.1 mm, 1.8 μm) column at 40 °C;. Injection volume was 1 μL. The mobile phase comprised 0.1% formic acid in water (A) and acetonitrile (B) at a flow rate of 0.3 mL/min. Gradient conditions were set as follows: 10% B (1 min), to 100% B (in 6 min), hold 100% B (4 min), to 10% B (in 0.1 min), and hold (1.9 min).

**Figure 1:**
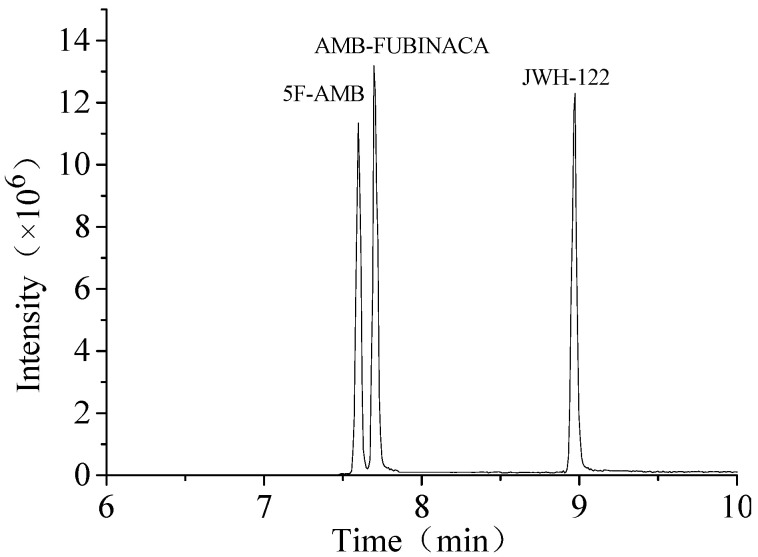
TIC of JWH-122, 5F-AMB, and AMB-FUBINACA.

The separated analytes were quantified using an AB SCIEX 6500^+^ quadrupole ion trap mass spectrometer (QTRAP MS) under the multiple reaction monitoring (MRM) mode with negative ion channels (Table 2). The mass spectrometry parameters were set as follows: curtain gas, 35 psi; collision gas, medium; ion spray voltage, 5500 V; source temperature, 550 ℃; nebulizing gas, 50 psi; heater gas, 50 psi. The HPLC-QTRAP-MS data were converted to SCIEX OS 1.4.0.18067 (SCIEX, Toronto, QUE, CA) for qualitative and quantitative analysis. The total ion chromatography (TIC) of the standard solution mixed with three synthetic cannabinoids was shown in Figure 1.

### 2.5. Method Validation

The method was validated in terms of extraction efficiency, matrix effect, selectivity, linearity, limit of detection (LOD), limit of quantitation (LOQ), recovery, and intra-day and inter-day precision. The lowest differed greatly from the highest concentrations of synthetic cannabinoids in the plasma and urine samples via pre-experiment. To ensure the accuracy, linearity was calibrated within the range of 0.04–2 ng/mL and 2–40 ng/mL in the plasma and 0.005–0.25 ng/mL and 0.25–5 ng/mL for urine samples. Sensitivity was expressed by the limit of detection (LOD) and limit of quantification (LOQ), which were evaluated according to signal (S) to noise (N) ratio with S/N ≥ 3 and S/N ≥ 10, respectively. Drug-free plasma samples were spiked with low (0.4 ng/mL), medium (4 ng/mL), and high (40 ng/mL) concentrations of mixed standard working solutions, while the urine samples were spiked with the concentrations of 0.05 ng/mL, 0.5 ng/mL, and 5 ng/mL of the three synthetic cannabinoids with six replicate analyses. The intra-day precision of plasma and urine samples was calculated in the same day, while inter-day precision was calculated by repeated analyses for six consecutive days. The recovery was evaluated by the peak area ratio of pre-extraction spiked samples against post-extraction spiked samples [17]. The matrix effect was evaluated by the concentrations of 0.1 ng/mL, 1 ng/mL, and 10 ng/mL in plasma and urine samples with three replicates.

### 2.6. Statistical Analysis

GraphPad Prism 7.0 was used for graphing, and the Statistical Package for Social Science (SPSS) version 19.0 was used for analyzing all experimental data by one-factor analysis of variance (ANOVA). All data were reported as the means or means with standard deviations (SDs) with triplicates. Differences were considered significant if *p* < 0.05.

## 3. Results

### 3.1. Extraction Conditions

In order to minimize the interference of substances in the matrices and increase recovery, the optimization of extraction conditions is essential [18]. The protein precipitation method is used to precipitate the protein and centrifuge the supernatant to determine the content of synthetic cannabinoids in biological samples. Organic reagents destroy structures of proteins in the plasma and precipitate the protein to extract synthetic cannabinoids from plasma. Thus, suitable sample preparation is important to reduce ion suppression of compounds in the matrices [13]. The matrix effect is critical in establishing reliable methods, and ignoring this effect may adversely affect the reliability of determination of analyte concentration [17]. The matrix effect is used as the evaluation parameter for the optimization of protein precipitation in plasma. A matrix effect value greater than one hundred percent indicates ion enhancement, whereas a matrix effect value less than one hundred percent indicates ion suppression [19]. Concurrently, recovery is used as an indicator for the extraction of synthetic cannabinoids in urine. Low recovery may be ascribed to incomplete extraction and elution of synthetic cannabinoids from the SPE cartridge.

**Figure 2:**
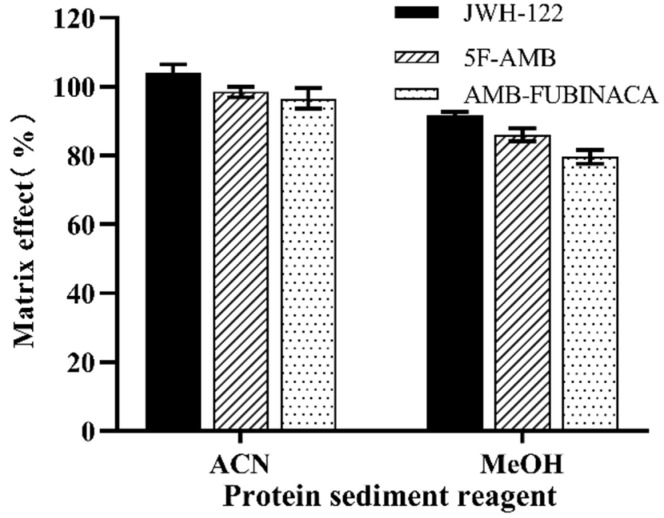
Effect of protein precipitation reagent on matrix effect.

**Figure 3:**
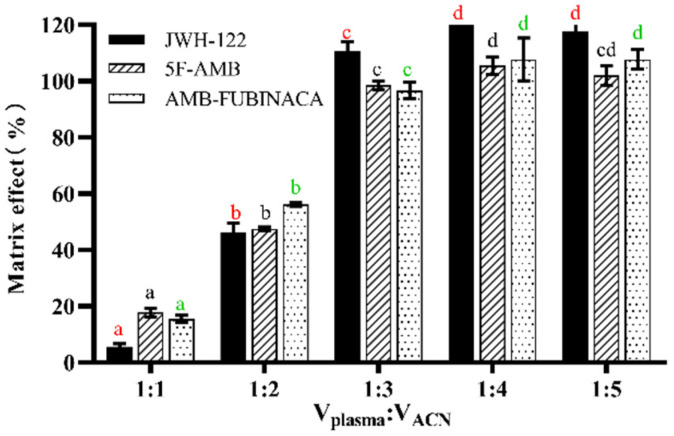
Effect of the volume of acetonitrile on the matrix effect. Any two volumes without the same lowercase letters in the same synthetic cannabinoid, as marked in the figure, indicate a significant difference of *p* < 0.05, n = 3, mean ± SD.

The present experiment was conducted to investigate the effects of methanol and acetonitrile on protein precipitation. As shown in Figure 2, when acetonitrile was used, the matrix effect values of the three synthetic cannabinoids were higher than with the use of methanol. Thus, acetonitrile was selected as the protein precipitation reagent. We also further explored the effect of different acetonitrile volumes on the matrix effect. The volume ratios of plasma to acetonitrile of 1:1, 1:2, 1:3, 1:4, and 1:5 were used to evaluate the matrix effect for three synthetic cannabinoids (Figure 3). Collectively, the matrix effect was significantly enhanced with the increasing volume of acetonitrile for each synthetic cannabinoid (*p* < 0.05), and the matrix effect value tended to be stable when the volume ratio of plasma to acetonitrile was 1:3. The matrix effect did not change significantly (*p* > 0.05) when the volume ratio of plasma to acetonitrile was greater than 1:3, as the volume of acetonitrile increased further. Finally, given the environmental protection and cost, the volume ratio of plasma to acetonitrile was selected as 1:3.

**Figure 4:**
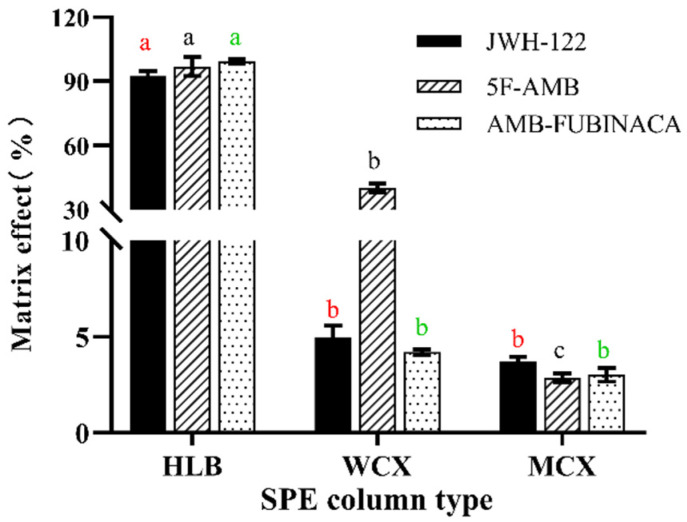
The effect of SPE column type on extraction recovery. Any two types without the same lowercase letters in the same synthetic cannabinoid, as marked in the figure, indicate a significant difference of *p* < 0.05, n = 3, mean ± SD.

Three different SPE cartridges, including the Waters Oasis HLB column (3cc/60 mg), Oasis WCX column (3cc/60 mg), and Oasis MCX column (3cc/60 mg), were harnessed for extraction. The Waters Oasis HLB column is a hydrophilic–lipophilic balanced extraction column, which is suitable for acid, alkali, and neutral compounds and thus can remove approximately 95% of matrix interferences (such as phospholipids, fats, salts, and proteins) in all biological matrices [20,21]. The Waters Oasis WCX (weak cation exchange) column provides high selectivity and recovery for strong bases and quaternary and withstands elution with high concentration solvents [22]. The Waters Oasis MCX, a mixed-mode cation-exchange sorbent, provides high selectivity and recovery for alkaline compounds [23]. The type of solid phase extraction column significantly affected the extraction recovery of the three synthetic cannabinoids (Figure 4). The extraction recovery with the Waters Oasis HLB column, which was 92.45 ± 2.29%, 96.95 ± 4.39%, and 99.33 ± 1.03% for JWH-122, 5F-AMB, and AMB-FUBINACA, respectively, was all significantly higher than the other two solid phase extraction columns (*p* < 0.05). Therefore, the Waters Oasis HLB column was finally selected for the extraction of these synthetic cannabinoids.

**Figure 5:**
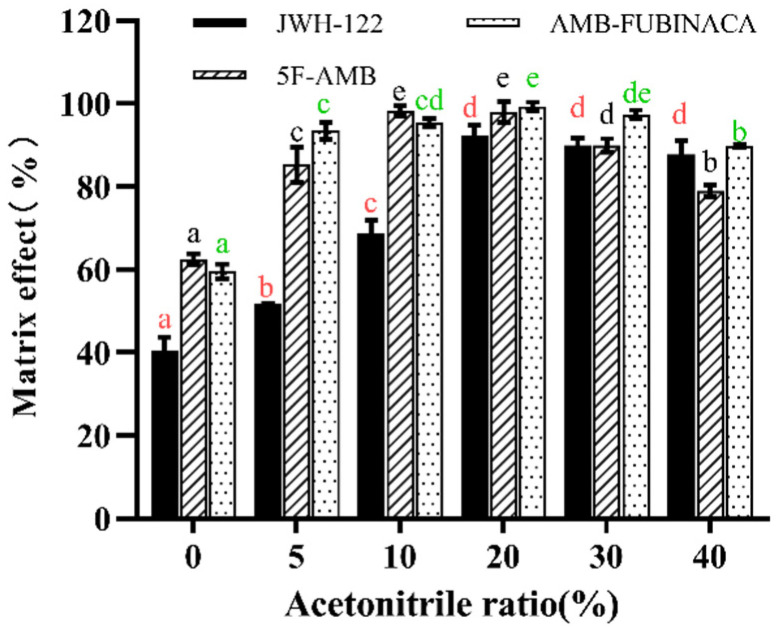
Effect of ratio of extract solution on matrix effect. Any two ratios without the same lowercase letters in the same synthetic cannabinoid, as marked in the figure, indicate a significant difference of *p* < 0.05, n = 3, mean ± SD.

Furthermore, we investigated the effect of the ratio of acetonitrile to water for the extraction recovery of synthetic cannabinoids in urine. Ultrapure water causes the incomplete transfer of synthetic cannabinoids into SPE cartridges. The addition of a certain volume of acetonitrile during extraction can effectively dissolve the synthetic cannabinoids. However, the synthetic cannabinoids are directly eluted by an SPE column under a high ratio of acetonitrile to water. Therefore, optimizing the ratio of the extract is crucial for the extraction of synthetic cannabinoids [13]. As shown in Figure 5, the total volume of the extract was maintained at 2 mL, while the ratio of acetonitrile to water changed. The volume ratio of acetonitrile to water was set to 0 (0 µL), 5% (100 µL), 10% (200 µL), 20% (400 µL), 30% (600 µL), and 40% (800 µL). As the proportion of acetonitrile increased, the recovery rate increased significantly for JWH-122 (*p* < 0.05). When the proportion of acetonitrile increased to 20%, the recovery rate stabilized and then no longer increased significantly (*p* > 0.05). Similarly, the recovery in the “20%” group was significantly higher than that in the “0”, “5%”, or “10%” groups for 5F-AMB (*p* < 0.05). Whereas the recoveries no longer increased significantly (*p* > 0.05) when the volume of acetonitrile continuously increased for AMB-FUBINACA, the recoveries in the “20%” group and the “30%” group were significantly higher than those in the other groups (*p* < 0.05). Therefore, the final volume of acetonitrile was 30% (600 µL) according to the optimum recoveries of the three synthetic cannabinoids.

**Figure 6:**
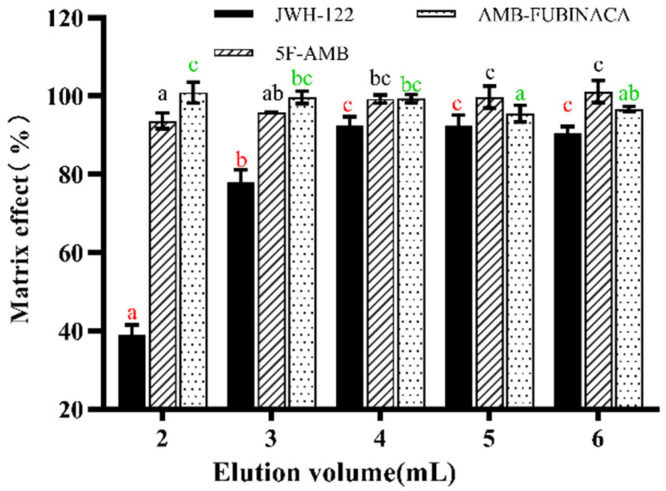
Effect of elution reagent volume on matrix effect. Any two elution volumes without the same lowercase letters in the same synthetic cannabinoid, as marked in the figure, indicate a significant difference of *p* < 0.05, n = 3, mean ± SD.

The influence of the volume of the elution reagent methanol on the extraction recovery of three synthetic cannabinoids was examined. The methanol (2 mL, 3 mL, 4 mL, 5 mL, and 6 mL) was harnessed for the complete elution of three synthetic cannabinoids by the SPE column. With the increase in methanol volume, the extraction recovery increased significantly for JWH-122 (*p* < 0.05) (Figure 6), and the administration of 4 mL of methanol reached the maximum recovery rate. For 5F-AMB and AMB-FUBINACA, the recoveries almost reached the maximum value when the volume of methanol was 4 mL. Therefore, 4 mL of methanol was employed by considering a high extraction recovery for synthetic cannabinoids.

### 3.2. Method Validation

**Figure 7:**
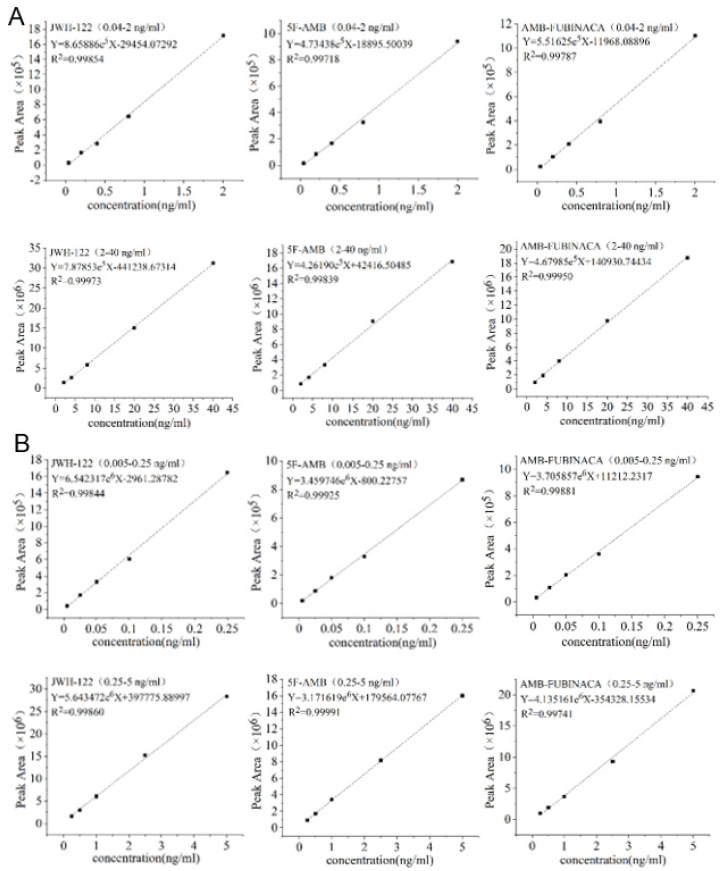
The matrix spike curves of JWH-122, 5F-AMB, and AMB-FUBINACA, (**A**) rat plasma, (**B**) rat urine.

All synthetic cannabinoids were confirmed to be linear within the calibration curve ranges (Figure 7 and Table 3). The correlation coefficients (R^2^) were more than 0.99. The LODs of synthetic cannabinoids covered the range of 0.003–0.004 ng/mL and 0.00125–0.002 ng/mL for plasma and urine samples, respectively. Concurrently, the LOQs of synthetic cannabinoids ranged 0.012–0.016 ng/mL and 0.003–0.005 ng/mL for plasma and urine samples, respectively (Table 3), which are lower than in previous studies (0.05–1.0 ng/mL and 0.1–1.0 ng/mL) [16,18,24,25]. The intra-day and inter-day precisions were 1.3–9.0% and 3.0–8.6% for plasma samples and were 2.8–6.7% and 3.9–8.8% for urine samples, respectively (Table 4).

The concentration of synthetic cannabinoid in the spiked matrix, these from A to I are 0.4, 4, 40, 0.05, 0.5, 5, 0.1, 1, 10 ng/mL.

The extraction recovery rate is used to evaluate the reproducibility of the extraction efficiency of the target analytes by the protein precipitation method and the solid phase extraction method [26,27]. The extraction recoveries of synthetic cannabinoids were 95.4–106.8% and 92–102% at three concentrations in plasma and urine samples, respectively (Table 4). Results from the matrix effect experiment are shown in Table 4, which fell in the ranges of 82–96% and 93–119% for plasma and urine samples, respectively. Slight ion enhancement was observed in urine, while less impact for the matrix effects was found in plasma. Taken together, the current method showed robust results of reproducibility and recovery rate and proved quite potent for the simultaneous analysis of synthetic cannabinoids in urine and blood samples.

### 3.3. Sample Analysis of Synthetic Cannabinoids

The LC-MS/MS method has been widely harnessed for detecting synthetic cannabinoids since 2014 [23]. The profiling methods should be continuously updated because new synthetic cannabinoids appear in the market all the time [28,29]. Therefore, the current study established a robust method for three emerging synthetic cannabinoids including JWH-122, 5F-AMB, and AMB-FUBINACA and confirmed the suitability of the methods via the plasma and urine samples of rats exposed to synthetic cannabinoids.

**Figure 8:**
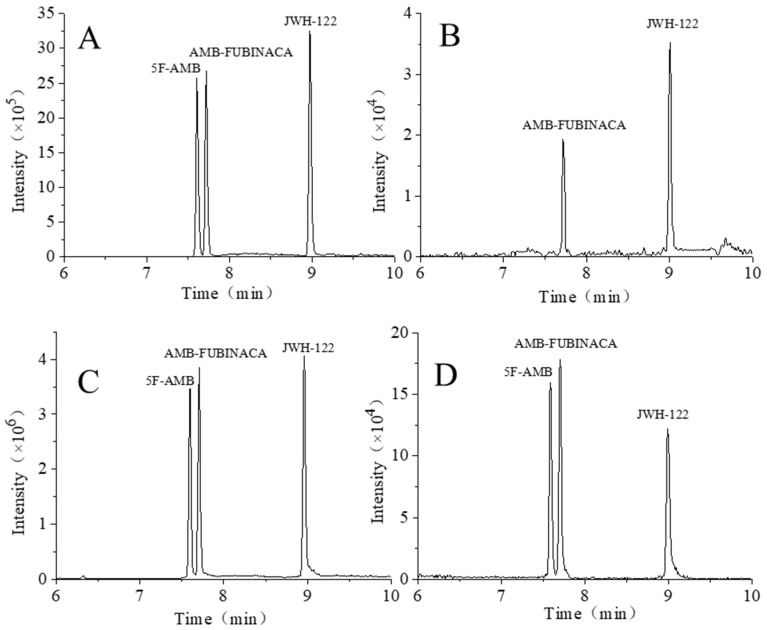
TIC of three synthetic cannabinoids of (**A**) matrix spike in rat plasma, (**B**) actual samples in rat plasma, (**C**) matrix spike in rat urine, and (**D**) actual samples in rat urine.

Figure 8 shows the typical chromatograms of both the blank sample and plasma and urine samples spiked with the synthetic cannabinoids. After 72 h of ingestion for three synthetic cannabinoids, AMB-FUBINACA and JWH-122 could be detected in plasma, while 5F-AMB was not detected. Concurrently, all synthetic cannabinoids could be simultaneously detected in urine. In detail, the concentrations of JWH-122 and AMB-FUBINACA were 0.08 ng/mL and 0.05 ng/mL, respectively, at 72 h in rat plasma. The relative standard deviation (RSD) values in all plasma samples were less than 20%, indicating qualified results. On the other hand, the concentrations of JWH-122, 5F-AMB, and AMB-FUBINACA were 0.20 ng/mL, 8.68 ng/mL, and 3.38 ng/mL, respectively, in rat urine after 2 h from oral gavage (Table 5). The concentrations of JWH-122, 5F-AMB, and AMB-FUBINACA then decreased to 0.12 ng/mL, 1.11 ng/mL, and 0.41 ng/mL, respectively, at 4 h. After 48 h, the concentrations of JWH-122, 5F-AMB, and AMB-FUBINACA attenuated to 0.02 ng/mL, 0.03 ng/mL, and 0.03 ng/mL, respectively, while the concentrations of JWH-122, 5F-AMB, and AMB-FUBINACA in rat urine were 0.01 ng/mL, 0.01 ng/mL, and not detected, respectively, at 72 h. The RSD values in all plasma samples were less than 20%, indicating credible results (Table 5). Collectively, the concentrations of the three synthetic cannabinoids gradually decreased during 0–72 h. The data revealed that a detection window of 2–3 days was achievable with low concentrations of JWH-122, 5F-AMB, and AMB-FUBINACA (the dose for the tail vein was 1 mg/kg) (Table 5). Our method is consistent or even superior with related studies, as samples could be stably profiled for 36 h or more with the detection concentration of 2–25 ng/mL and as it achieved the detection of three synthetic cannabinoids with lower content [18].

## 4. Conclusions

In the current study, we established and validated an LC–MS/MS method for the simultaneous determination of three synthetic cannabinoids in rat plasma and urine. We optimized the conditions of protein precipitation and solid phase extraction. The precision and recovery of the method were 1.3–9.0% and 95.4–106.8% for plasma samples and 2.8–8.8% and 92–102% for urine samples, and the actual rat biological samples could be effectively detected within the linear range. In summary, the current study obtained an environmentally friendly, simple, efficient, and low-consumption pretreatment method of synthetic cannabinoids with satisfactory linearity, precision, and recovery and beneficial sensitivity. The validated method was successfully applied to determine the concentration of WH-122, 5F-AMB, and AMB-FUBINACA in the plasma and urine samples of rats orally administered with synthetic cannabinoids. Our study establishes an efficient method for the detection of synthetic cannabinoids and facilitates the prevention and control of new synthetic cannabinoids.

## Figures and Tables

**Table 1:** A. Parameters for protein precipitation. B. Parameters for solid phase extraction.

(A)	
Factor	Type of protein precipitation reagent
1	Acetonitrile
2	Methanol
Factor	Volume of acetonitrile to plasma
1	1:1
2	1:2
3	1:3
4	1:4
5	1:5
(B)	
Factor	Type of SPE column
1	Oasis HLB
2	Oasis WCX
3	Oasis MCX
Factor	Proportion of extract solution (%)
1	0
2	5
3	10
4	20
5	30
6	40
Factor	Elution reagent volume (mL)
1	2
2	3
3	4
4	5
5	6

**Table 2:** MRM transitions and conditions of each analyte.

Compound Name	Precursor Ion (*m/z*)	Product Ion A (*m/z*)	Product Ion B (*m/z*)	DP (*V*)	CE (*V*)
JWH-122	356.2	169.2	214.2	32/32	176/177
5F-AMB	364.2	233.2	304.2	32/21	72/71
AMB-FUBINACA	384.2	253.2	324.2	30/23	80/61

Product ion A is the quantitative ion, product ion B is qualitative ion. DP is declustering potential, CE is collision energy.

**Table 3:** The linear range and correlation coefficient (r2) of JWH-122, 5F-AMB, and AMB-FUBINACA (n = 6).

Compound Name	Linear Range (ng/mL)		Correlation Coefficient		LOD (ng/mL)		LOQ (ng/mL)	
	Plasma	Urine	Plasma	Urine	Plasma	Urine	Plasma	Urine
JWH-122	0.04–2	0.005–0.25	0.99854	0.99844	0.003	0.0003	0.012	0.00125
	2–40	0.25–5	0.99973	0.99860				
5F-AMB	0.04–2	0.005–0.25	0.99781	0.99925	0.004	0.0005	0.016	0.002
	2–40	0.25–5	0.99839	0.99991				
AMB-FUBINACA	0.04–2	0.005–0.25	0.99787	0.99881	0.004	0.0004	0.012	0.0015
	2–40	0.25–5	0.99950	0.99741				

**Table 4:** The intra-day and inter-day precision, recovery, and matrix effect of JWH-122, 5F-AMB, and AMB-FUBINACA (n = 6).

Compound Name	Intra-Day Precision (%)						Inter-Day Precision (%)						Recovery (%)						Matrix Effect (%)					
	Plasma			Urine			Plasma			Urine			Plasma			Urine			Plasma			Urine		
	A	B	C	D	E	F	A	B	C	D	E	F	A	B	C	D	E	F	G	H	I	G	H	I
JWH-122	9.0	5.9	7.6	4.0	4.5	2.8	8.0	8.1	3.8	8.1	5.4	5.5	96.5	100.2	102.5	95.1	98.7	101.6	95.1	95.9	92.8	118.0	93.4	97.6
5F-AMB	6.4	3.1	1.5	3.8	3.8	3.1	8.6	6.4	4.2	6.7	4.9	3.9	98.7	97.6	95.4	92.0	100.4	96.6	89.1	86.0	88.5	113.6	99.4	95.7
AMB-FUBINACA	2.3	3.4	1.3	6.7	6.1	4.4	4.6	4.4	3.0	7.8	6.7	8.8	106.8	102.3	98.2	97.9	98.3	102.0	93.9	82.6	92.2	101.0	95.4	96.6

**Table 5:** Determination of JWH-122, 5F-AMB, and AMB-FUBINACA in rat plasma and urine.

Compound Name	Plasma		Urine											
	72 h		2 h		4 h		8 h		24 h		48 h		72 h	
	Concentration (ng/mL)	RSD (%)	Concentration (ng/mL)	RSD (%)	Concentration (ng/mL)	RSD (%)	Concentration (ng/mL)	RSD (%)	Concentration (ng/mL)	RSD (%)	Concentration (ng/mL)	RSD (%)	Concentration (ng/mL)	RSD (%)
JWH-122	0.08	4.38	0.20	5.51	0.12	8.66	0.09	7.60	0.06	14.36	0.02	16.36	0.01	13.22
5F-AMB	ND	/	8.68	9.96	1.11	9.17	0.72	9.61	0.14	6.08	0.03	12.20	0.01	13.86
AMB-FUBINACA	0.05	8.42	3.38	4.39	0.41	10.06	0.19	3.92	0.10	12.80	0.03	14.45	ND	/

## Data Availability

Not applicable.

## Abbreviations

Abbreviations and their full names in the article are shown below:

NPS	New Psychoactive Substance
EWS	Early Warning System
EMCDDA	European Monitoring Centre for Drugs and Drug Addiction
JWH-122	4-methylnaphthalen-1-yl-(1-pentylindol-3-yl) methanone
5F-AMB	methyl (1-(5-fluoropentyl)-1H-indazole-3-carbonyl)-L-valinate
AMB-FUBINACA	methyl 2-(1-(4-fluorobenzyl)-1H-indazole-3-carboxamido)-3-methylbutanoate
LC-MS/MS	liquid chromatography tandem mass spectrometry
JWH-018	1-Pentyl-3-(1-naphthoyl) indole
AKB-48	N-(1-Adamantyl)-1-pentylindazole-3-carboxamide
MDMB-CHMINACA	indole carboxamides
LOD	limit of detection
LOQ	limit of quantitation
